# Country Case Studies in Economic Fitness: Mexico and Brazil

**DOI:** 10.3390/e20100753

**Published:** 2018-10-01

**Authors:** Kirstin Roster, Luciana Harrington, Masud Cader

**Affiliations:** International Finance Corporation, World Bank Group, Washington, DC 20433, USA

**Keywords:** economic complexity, public policy, development economics, economic fitness, development strategy, industrial diversification

## Abstract

We leverage a new complexity framework called Economic Fitness, which characterizes an economy’s level of diversification and its capabilities to produce more complex products. It can be used to predict economic growth and competitiveness. This paper describes an application of Economic Fitness called the Country Opportunity Spotlight (COS) that assesses a country’s current level of capabilities and demonstrates which industries have upgrade and diversification potential given those capabilities. It helps unlock the explanatory and predictive power of Economic Fitness for policymakers. COS results serve as a starting point for policymakers to shape and validate priorities, compare countries, asses the capabilities needed in specific industries and begin identifying constraints to growth. We showcase the use of this framework for Mexico and Brazil. These countries provide an interesting case study, as they have similar growth outlooks yet demonstrate different productive capabilities. Examining Mexico and Brazil side by side illustrates the value this analysis can have on deciphering structural change and decision making and at the same time reinforces the need for a nuanced consideration of each country’s unique context.

## 1. Introduction

Evidence-based decision making is increasingly important for policymaking in international development. The United Nations recognizes the power of data and evidence in achieving the Sustainable Development Goals (SDGs) [1,2,3] and supports innovative approaches in addressing critical development challenges (UN Initiatives and partnerships for the use of data science for development include UN Global Pulse, GSMA Big Data for Social Good Initiative and the Data Revolution Group.). Economic Fitness responds to the need for evidence in policymaking, by providing a rigorous quantitative basis to develop strategies for growth.

Economic Fitness is a quick and cost-effective analytical approach that enables immediate insights. It does not require time- and resource-consuming surveys but instead is based on existing data [4]. It assesses country capabilities and is predictive of long-term growth in GDP per capita [5,6] as well as competitiveness in over 4000 different goods and services [7].

Economic Fitness has many applications to the policy process. We introduce a framework called the Country Opportunity Spotlight (COS) [8], which helps unlock the predictive and explanatory power of the Economic Fitness toolkit. This paper demonstrates how the methodology can be of value to practitioners aiming to achieve economic growth in emerging economies. The COS helps policymakers assess country capabilities and filters for opportunities that leverage these capabilities. Furthermore, the COS can inform planning decisions to reduce constraints to growth and support innovation, diversification and capability upgrade.

This paper uses the case studies of Mexico and Brazil to show how the Economic Fitness toolkit can be applied in practice. We illustrate the similarities between Mexico and Brazil in terms of their Fitness and predicted future growth trajectories and demonstrate how key differences between the countries are expected to lead to unique growth pathways. Brazil and Mexico, while similar at an aggregate scale, have reached their current level of development on different trajectories utilizing different underlying sets of capabilities. Using the transport equipment sector as an example, the paper shows select results of the COS opportunity filter. In the discussion, we highlight the importance of incorporating aspects of the country context.

The rest of the paper is organized as follows: Section 2 illustrates the contribution of Economic Fitness to policymaking. Section 3 describes the Economic Fitness toolkit and COS framework. Section 4 presents sample results for the country cases of Mexico and Brazil. Section 5 provides a summary and discussion.

## 2. Value of Economic Fitness for Public Policy

Economic Fitness has important applications at the country level. The predictive power of Economic Fitness to GDP is one that policymakers and other stakeholders (such as industry associations, private companies, CSOs and citizens) interested in economic growth and development should care about. Economic growth matters not only as a measure of income but also as a broader indicator of access to and availability of services such as health care and education. The field of development economics has elaborated various and sometimes divergent theories to explain growth [9,10,11,12,13,14,15,16], as well as explain how it can improve livelihoods and well-being [17,18] (This is a non-exhaustive list but rather seeks to illustrate a few examples.). Economic Fitness contributes to the literature by allowing us to examine the building blocks of economic growth in a given country. It lets us dissect the industries and sectors that can generate growth with existing endowments. By knowing the building blocks, policymakers can create environments conducive for market players to construct pathways for greater and more inclusive economic development.

Economic Fitness thus matters to policymakers. More Economic Fitness means more growth. It also means more development: more skills, knowledge, ideas and opportunities for people. Greater levels of Economic Fitness give a larger capacity at the country level to produce many and more complex products. A country with greater levels of Fitness can do more unique, specialized things that similar countries with lower levels of Fitness cannot do.

Using applications from Economic Fitness such as the Country Opportunity Spotlight (COS), introduced in Section 3 gives policymakers tangible and actionable indications to determine how new pathways for growth may develop. The COS serves as a strength-finder by identifying industries that are likely to promote growth by leveraging a country’s endowments. We argue that knowing which industries can leverage current endowments—such as infrastructure and skills—allows practitioners to identify key bottlenecks constraining growth. Faced with resource constraints and competing priorities, it is not always clear to policymakers which barriers are most critically hindering growth and therefore which policy could have the largest impact. Economic Fitness does not give a complete answer either but it provides a useful first filter and invites exploration.

## 3. A Framework for Decision Makers

### 3.1. Economic Fitness Toolkit in Light of Its Applications

*Economic Fitness* is a country level metric based on the diversification and complexity of a country’s competitive exports. As countries industrialize, they increase their Fitness, moving along the product progression to more complex industries [4,19]. Economic Fitness is predictive of long-term growth in GDP per capita [5,6] and gives an indication of possible poverty and middle-income trap behaviors [20]. It complements output-based measures such as GDP, because it focuses on the underlying capabilities available that define a country’s potential for growth *Capabilities* is a broad term for all the endowments that influence a nation’s ability to produce goods and services, such as physical inputs, infrastructure, human capital, technology, or governance structures.

Because of its predictive power, raising Fitness can lead to greater GDP per capita. The two dimensions to increasing Fitness are diversification and upgrade to more complex industries. The *product progression network* lets us understand the successful pathways to growth of other countries and *progression probability* helps assess the likelihood of competitiveness in industries within the next five years. These metrics model how other countries have successfully upgraded and diversified to new industries [19]. They give an indication of which industries a country will likely diversify into, given its existing productive structure [7]. We also refer to the progression probability as a country’s *feasibility* of a given industry.

The *complexity of industries* assesses the level of capabilities needed to competitively export a product. Countries with high Fitness are able to produce complex goods and services. Diversification to more complex industries can help raise Fitness and build capabilities that may lead to new opportunities in the future.

**Figure 1:**
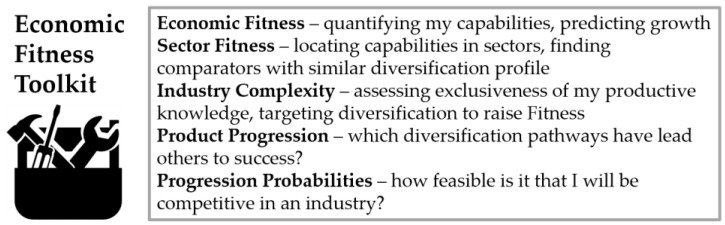
The tools in the Economic Fitness toolkit have different applications for policymakers and other stakeholders. The metrics in the toolkit are explained further in the Glossary in Appendix A, the technical definitions in Appendix D, as well as in the cited literature. Data sources are described in Appendix C.

These three instruments (Economic Fitness, Industry Complexity, Progression Probability) together form the foundation of the *Economic Fitness toolkit* (Figure 1), a collection of rigorous techniques to model the dynamics of the global economy. Used in combination, these tools help provide a filter for diversification and capability upgrade opportunities.

### 3.2. Country Opportunity Spotlight

**Figure 2:**
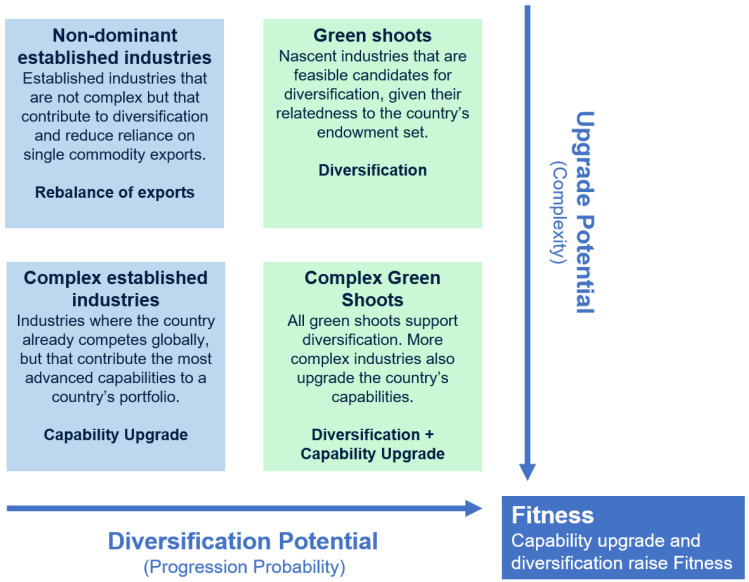
Country Opportunity Spotlight (COS) Opportunity Typology. This figure illustrates the different types of opportunities identified by the COS framework. The two key opportunity dimensions reflected on the axes mirror the two dimensions of Economic Fitness: Diversification and complexity of exports. Opportunities with high progression probability (*x*-axis) promote a country’s diversity of production. High complexity upgrade potential (*y*-axis) captures industries that upgrade productive capabilities. The typology further distinguishes between established (Revealed Comparative Advantage > 1) and green shoot industries, resulting in four types of opportunities, each aimed at different policy outcomes.

In this section, we introduce a new framework called the Country Opportunity Spotlight (COS), which uses the tools of Economic Fitness to identify growth pathways a country is likely to follow, given its set of endowments. (In the development of the COS methodology, the authors tested other economic complexity approaches [9]. Besides technical improvements and mathematical robustness of Economic Fitness, which are discussed in the literature, we find that the Economic Fitness toolkit provides intuitive results with high interpretability and enables forward-looking applications). Based on a country’s individual capability profile, the COS provides a filter of goods and services that are: (i) feasible because the country already possesses many of the required capabilities and (ii) likely to upgrade the existing capabilities. These industries have the potential to contribute to a country’s future Fitness and consequently affect GDP per capita. These industries are likely to grow, unless there are constraints to growth. The COS lets policymakers better gauge probable future development and identify barriers constraining growth. To match varying policy priorities, the COS distinguishes between different kinds of industries, a typology of which is presented in Figure 2. (To identify these opportunity types, the COS utilizes metrics on industry complexity, feasibility of progression, growth relative to global industry rates, industry size, revealed comparative advantage, the product progression taxonomy, Sector Fitness and other data points as needed. These variables arise from the Economic Fitness toolkit outlined above and are described in more detail in Appendix A.)

The COS opportunity filter is country-specific. It selects industries that build on available productive knowledge. The opportunity typology leaves the policymaker flexibility to adjust to country context and planning needs, depending on factors such as:

- the policymakers’ planning horizon. Nascent industries will take time to develop and are more viable in five- to seven-year plans or longer-term visions, while established industries can be developed in shorter time periods.
- strategic goals beyond pure economic growth. The COS filter lists industries that likely support economic growth, though economic and social development is multi-faceted and governments may support several goals simultaneously. For example, if the policymaker wants to achieve more inclusive growth, the results can be further filtered by their potential to create opportunities for minority groups or in specific geographic regions. Table 1 above provides examples.
- alignment with planned interventions. For example, a planned road could benefit several industries. It can attract investors to new markets, making some green shoots much more feasible than others.

While the COS does not prescribe certain policies or the role of government in growth, the COS Opportunity Typology can support a variety of policy objectives, for example: (i) Industrial growth: expanding established industries for additional growth, (ii) Green shoot development: supporting industries that are growing but have not yet been established and are typically newer or more specialized industries, (iii) Industrial Capacity and Knowledge Building: incentivizing development of innovative capacity and skills, as a way to boost R&D, innovation and economic resilience and (iv) Export Driven Development: leveraging their natural endowments but with low value added. This generally refers to primary goods and agriculture.

A key advantage of the Economic Fitness toolkit is its multi-faceted scaling power. Country capabilities are assessed globally as well as at the sub-national level. Industry opportunities and competitiveness are captured by sector, sub-sector and even detailed goods and services that distinguish over 4000 categories. This means that the policymaker can use the COS framework as an early stage assessment tool and strategy guide at various levels.

### 3.3. Limitations

Economic Fitness is not a single solution for policymaking. Instead, it should be complemented by additional information and the policymaker’s judgment. As a zero-parameter approach, Economic Fitness by definition does not make explicit the capabilities that are critical to development. Specifically, it does not tell us whether there is a suitable governance framework, adequate access to road and port infrastructure, market demand for products and whether the skills necessary to develop specific products are mobile and readily available. Policymakers will have to navigate the current socio-political, economic and industrial landscape as they begin the policy process. Therefore, the COS is a first filter that will need to be followed by additional information.

Economic Fitness analytics can be complemented by an explicit assessment of country capabilities and barriers to growth. A 2014 McKinsey report, for instance, aims to make explicit some of the factors listed in Table 1 that are constraining Brazil’s growth, such as low R&D investments and market distortions [21]. Such information not only helps narrow the industry filter but also creates a roadmap to implementing the COS results. It takes the policymaker from “Which industries can promote growth?” to “How can our policies achieve growth?”. Growth Diagnostics is another example of a useful complementing approach [22]. Economic Fitness at the sub-national level assesses the geographic position of country capabilities and can provide other valuable insights [23].

The next section provides sample results of the COS for Mexico and Brazil. Economic Fitness in its most aggregate form shows similar starting conditions in the next stage of the countries’ development. In spite of this, Mexico and Brazil have differing capability profiles and opportunities for growth and consequently, may require different policy responses.

## 4. Mexico and Brazil

Brazil and Mexico are the largest emerging markets in Latin America and the 8th and 15th largest economies in the world, respectively. They each carry a gross domestic product (GDP) in the trillions with Brazil’s economy reaching 1.8 trillion and Mexico’s reaching 1.0 trillion in 2017 (Using the World Bank Group Atlas Methodology). Despite being large economies, Mexico and Brazil have developed in a highly unequal fashion with wealth concentrated at the top of the income scale. Both countries have relatively high GINI coefficients, a measure of income inequality, with Brazil’s GINI reaching 0.51 and Mexico’s at 0.48, far below the OECD average of 0.31. In addition, both economies fall below the upper middle-income threshold (As defined by the World Bank at $12,056 in 2018) of GDP per capita of $12,056 as defined by the World Bank, with Mexico’s GDP per capita at $8201 and Brazil at $8650 in 2017. Lastly, Mexico and Brazil host a combined population of 334 million: 206 million in Brazil and 128 million in Mexico [24]. Please see Appendix B for additional economic comparisons. 

### 4.1. Similar Growth Outlook

**Figure 3:**
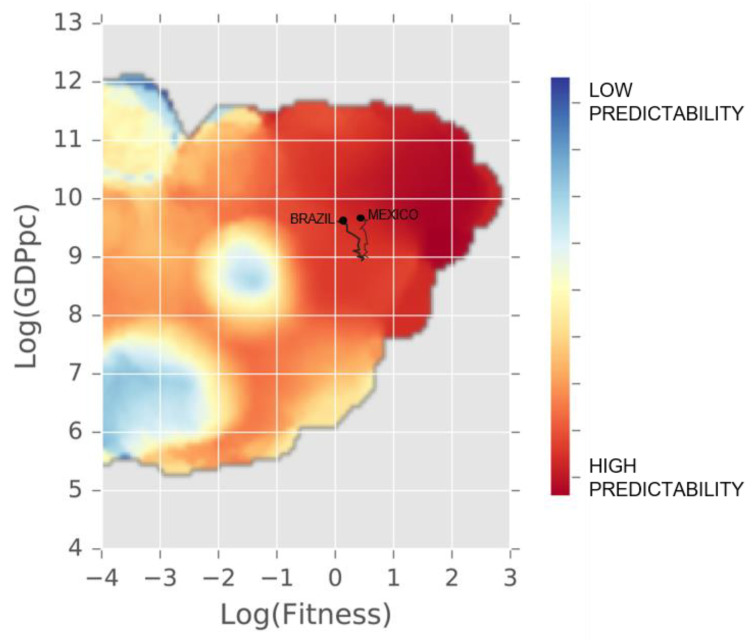
Fitness-GDPpc plane. Mexico and Brazil’s position in the Fitness-GDPpc plane in 2015 and their historical development trajectories since 1995. Mexico and Brazil occupy a very similar position of the Fitness-GDPpc plane. The color shading indicates predictability of future development. Countries in dark red regions follow the most predictable pathways. Data: [5,24,25].

**Figure 4:**
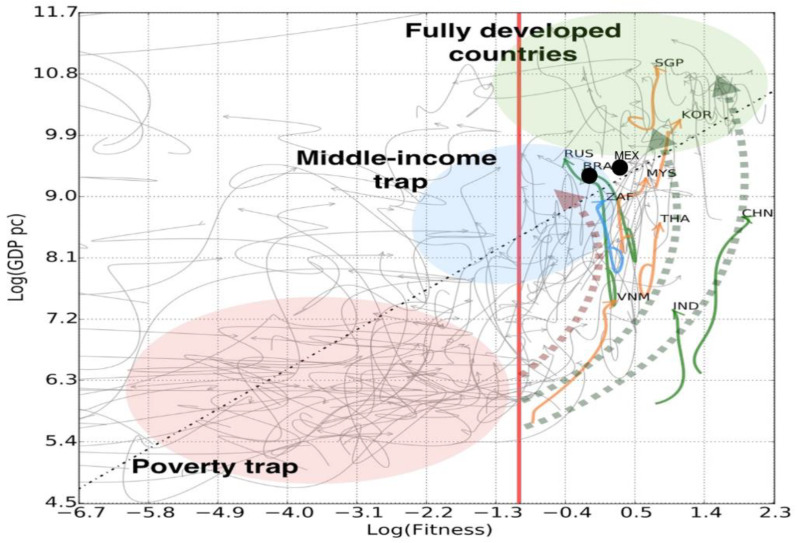
Dynamics in the Fitness-GDPpc plane. This chart represents the same space as Figure 3 and highlights the trapping and escape dynamics in the Fitness-GDPpc plane [18]. Historical trajectories (grey lines) illustrate how countries in the poverty and middle-income trap regions are unable to raise income. The colored trajectories represent examples of successful escape from the traps: increased Fitness followed by rising GDP per capita. Mexico and Brazil are located near the middle-income trap barrier. Data: [24,25].

Economic Fitness tells us that Mexico and Brazil face very similar starting conditions for the next stage of their development pathway. They are expected to raise GDP per capita in the next five years and face the challenge of avoiding the middle-income trap [10]. These predictions stem from analysis of how countries’ Fitness relates to their wealth, which is characterized by the dynamics of the Fitness-GDPpc plane (Figure 3 and Figure 4). This section provides details of these results. 

The Fitness-GDPpc plane defines a space to capture growth dynamics beyond a country’s level of income. This space allows us to predict long-term growth in GDP per capita, by examining the past trajectories of all countries globally. Quite intuitively, some countries’ future pathways are easier to predict than others. The space quantifies predictability in different areas of the plane. Grouping countries by their predictability regimes improves growth forecasts not only in this model but also for other econometric approaches, such as the IMF World Economic Outlook [5,6].

Brazil and Mexico occupy nearly the same position in the Fitness-GDPpc plane (Figure 3). They have a positive outlook for future growth and we expect both Brazil and Mexico to raise GDP per capita in the next five years (5.8 percent and 5.5 percent growth respectively between 2015 and 2019) (We learn this from examining other countries in this position of the plane and note that they followed relatively stable and predictable pathways. Malaysia, for example, was in a very similar point of the plane around 2005 and has since grown both GDP per capita and Fitness. Malaysia is now in an even more predictable space of the plane with GDP per capita expected to continue its upward trajectory.) The countries are in a relatively predictable area of the plane. Besides providing high confidence to predictions, this means that there is lower risk to long-term growth in GDP per capita than may be expected from examination of economic indicators alone. Short-term growth may be affected by short-term volatility but long-term growth is likely to be relatively stable in this region of the plane. [5].

Examination of the Fitness-GDPpc plane also helps identify a two-dimensional barrier of the poverty trap, which is usually defined only by an income metric, such as GNI per capita (The 2018 middle income range is $3956–12,235 GNI per capita, as defined by the World Bank.). This single-metric view alone cannot explain how some countries are able to grow robustly, while others are trapped. The Fitness-GDP per capita plane helps shed light on the dynamics that allow countries avoid the trap: after reaching a specific level of GDP per capita, countries are either able to build Fitness and begin a successful exit or remain in the trap [20].

These dynamics illustrate that current levels of Economic Fitness will keep both Mexico and Brazil within the boundaries of the middle-income trap (Figure 4). The middle-income trap is important as it describes extended stagnation in economic growth at a defined level of GDP per capita. Yet the trap is not inevitable, as there are many successful cases of escape, particularly among the phenomena of the East Asian tigers that consistently maintained high levels of economic growth since the 1960s fueled by exports and rapid industrialization. Economic Fitness can give clues for readiness to escape the trap, allowing for sustained growth in GDP per capita [20].

Though they occupy the same region of the plane, Mexico and Brazil took different historical trajectories to reach this position (Figure 3), which points to a fact observable across countries globally: there are different ways to get to the same level of Fitness and wealth and different places to go from the same starting conditions. In fact, Brazil and Mexico began at nearly the same position of the Fitness-GDPpc plane in 1995. Since then, Mexico maintained its level of capabilities, while Brazil lost Fitness.

Exiting the risky middle-income trap region of the plane will depend on Mexico and Brazil’s ability to build capabilities that can help grow income. For Brazil, this means reversing its trend of deteriorating Fitness. Mexico is only in a slightly better position and capability development will be critical to move the country into the next stage of development. For deeper clues about how to improve fitness, we can look at the sectoral composition of the countries’ capabilities.

### 4.2. Many Different Pathways to Raise Fitness

Fitness is determined by two factors: diversification and complexity. Raising Fitness means upgrading capabilities (doing more complex things) or broadening the portfolio (doing more diverse things), or both. In assessing whether a country is on track to achieve this, it is helpful to examine capabilities at a sectoral level. Understanding a country’s capability profile can shed light on the set of industries that are within reach. This is based on the fundamental idea that diversification is not random but instead a natural, gradual process in which countries develop their capabilities until they have the mix that is needed to compete in a given industry. If a country already has most of the skills and technologies needed to produce, say, computers, then entering that market will be more feasible than an industry where most capabilities would have to be developed or imported. Because of this, a country’s capability mix can give an indication of likely diversification and upgrade opportunities [4].

**Figure 5:**
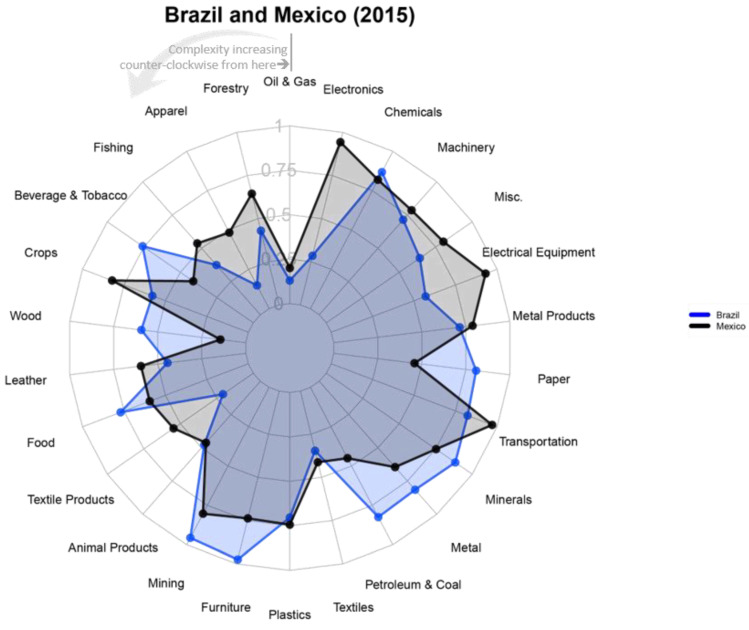
Sector Fitness. Mexico and Brazil differ in their sectoral capabilities, as is demonstrated by their normalized sector fitness rank in 2015. This measure ranges from the global leader (1) to the lowest performer (0). It is computed by applying the Fitness algorithm to a subset of the total universe of products, grouped into sectors using the North American Industry Classification System (NAICS). Details on the sector fitness algorithm can be found in Appendix D. Sectors are ordered counter-clockwise by their complexity. Data: [25].

While Mexico and Brazil have a similar aggregate level of Fitness (Figure 3), the countries draw their capabilities from somewhat different sectors, as is illustrated in Figure 5. Even though Mexico has a slightly higher overall Fitness, there are several sectors, where Brazil is stronger, such as animal products, chemicals, mining, or food production. Both countries enjoy a competitive global position in complex manufacturing sectors, such as transportation equipment or metal processing. Mexico has higher Fitness in more complex goods such as electronics, machinery and electrical equipment. These industries can represent opportunities for the private sector to retain a strong global position, by expanding to new markets, increasing value addition or upgrading to very sophisticated products and services.

**Figure 6:**
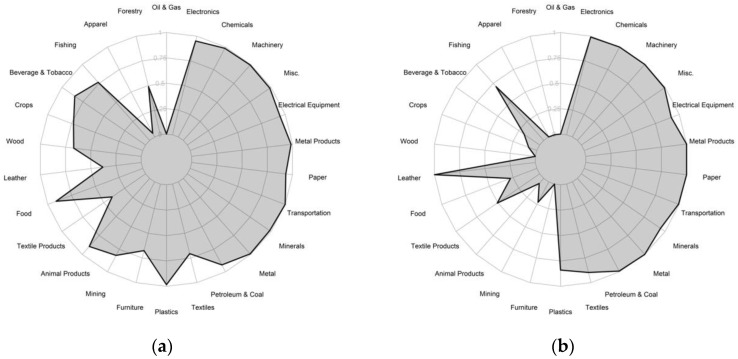
Sector Fitness gives clues to diversification strategies. Germany and Japan follow different diversification strategies, as can be seen from their sector fitness in 2015. Panel (**a**) shows Germany’s broad diversification pattern with strong performance in many different sectors. Panel (**b**) contrasts this with Japan’s sector fitness, which is focused on high-complexity sectors on the right side of the radar plot (e.g., electronics) and traditional industries (e.g., fishing). Data: [25].

Countries have achieved high Fitness in different ways and there is no single strategy for development. Germany and Japan, for example, both rank in the top five highest Fitness countries in 2015. But their capabilities stem from a very different diversification strategy (Figure 6). Germany is competitive in many sectors, including very complex industries such as electronics and chemicals but also in more basic commodities including food and animal products. Japan instead focuses on only high-complexity sectors (right half of the radar plot) together with select industries of cultural significance, such as fishing. Economic Fitness and the COS do not provide a prescription to growth or a framework for industrial policy. Instead, the COS aims to identify the capabilities that led to a country’s development in the past and then predict which industries should emerge from these capabilities. It emphasizes a country’s comparative advantage as an indicator of its potential. It is then up to policymakers to determine which barriers are preventing an ecosystem for growth.

The example of Mexico and Brazil shows that capabilities are country-specific. While Mexico and Brazil have a similar aggregate level of Fitness, their productive knowledge is localized in different sectors. Capabilities enable the production of a country’s unique mix of goods and services. They also determine how easy it is for a country to enter new industries. The Country Opportunity Spotlight (COS) filters the most feasible and complex industries for Mexico and Brazil. The next section illustrates a sample of COS results in the transport equipment sector.

### 4.3. Opportunities Arise from Capabilities: Examining Mexico and Brazil’s Transport Equipment Industries

Mexico and Brazil have historically strong machinery manufacturing sectors. In the case of Mexico, the manufacturing sector benefitted heavily from trade liberalization under NAFTA. In addition, the industry took advantage of integration into global value chains and developed linkages with universities and state governments [26]. In the case of Brazil, the sector benefitted from traditional import substitution industrialization starting in the 1950s and more recently has benefitted from tax breaks and special export zones. The sector has been losing competitiveness over time, however [27].

The strength of Brazil and Mexico’s transport equipment manufacturing sectors shows up in sector fitness, where both perform in the top quarter of all countries globally (see Figure 5). Their unique endowments define which upgrade and diversification strategy is feasible and likely to promote growth.

Brazil remains a competitive exporter of aircraft, though volumes declined by an average 7 percent annually between 2012 and 2015. Globally, this industry grew 8 percent annually in this timeframe, indicating that Brazil falls behind global growth rates by over 15 percentage points. Similar patterns are observable in most of Brazil’s large transport equipment export products. In fact, Brazil recorded an overall Fitness decline in transport equipment, falling from 17th place in 2000 to a global rank of 25 in 2015 (Figure 5).

In spite of this trend, Brazil has opportunities to recover its Fitness in transport equipment manufacturing. Its most complex competitive industries that are still growing include railway and track equipment as well as bus bodies. Given its capabilities in aerospace, there is also potential for new industries to emerge, such as aircraft parts. A sample of Brazil’s opportunity filter is listed in Table 2.

**Figure 7:**
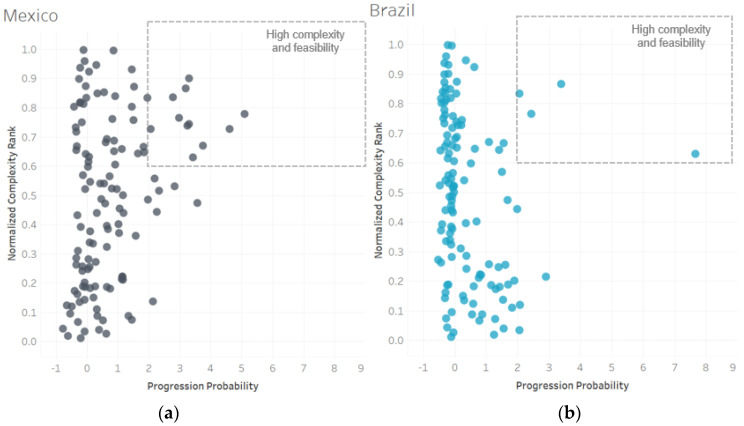
Feasibility in transport equipment. Products in the transport equipment sector are represented by their normalized industry complexity rank (*y*-axis) and a country-specific measure for the progression probability of Mexico and Brazil (*x*-axis). Industry complexity is normalized, so that the least complex product in transport equipment has a normalized score of 0 and the most complex has a score of 1. Progression probability is normalized so that zero represents the average likelihood of countries of having RCA >1 in an industry, a positive score represents above average likelihood and a negative score below average likelihood. Panel (**a**) shows Mexico’s feasibility (progression probability) of achieving and maintaining competitiveness in each transport product. There are several complex industries with high progression probability scores, indicating possible capability upgrade opportunities. Panel (**b**) shows Brazil’s feasibility in the same industries. There are fewer complex industries with high progression probability scores, though progression is relatively feasible in lower-complexity transport products. Data: [7,25].

Feasibility of expansion and retention of competitiveness in transport equipment tends to be lower for Brazil than Mexico (Figure 7). Feasibility is measured using Progression Probability and is based on empirical evidence about the successful diversification pathways of other countries globally. It captures the likelihood of becoming competitive in the export of a specific good or service. Mexico has very high feasibility scores in complex products, while Brazil’s most feasible goods are some of the least complex in the sector. This means that Mexico’s capabilities build a strong base for complex transport products, both to strengthen established industries and develop new niches. Brazil’s capabilities also give rise to complex transport goods. It just has lower progression likelihood in these industries, meaning the barriers to reach competitiveness may be higher and more capabilities need to be developed before becoming competitive.

Mexico has a relatively established motor vehicle parts industry with high export volumes and global competitiveness. Given the high complexity of some of these motor parts, this industry represents an opportunity for capability upgrade in Mexico. At the same time, Mexico can leverage its strength from existing industries to move to new products, such as trucks and buses (see Table 3). These have lower complexity than auto parts but are relatively feasible for Mexico and can help diversify the country’s portfolio.

Policymakers can use these results to understand likely developments of the transport equipment sectors in Mexico and Brazil and to assess the feasibility of policies such as engineering skills training or transport infrastructure.

Economic Fitness can serve as a policy guide, though development strategies would need to consider current capabilities, together with market demand factors, endowments and political will, infrastructure networks and other potential impediments to growth.

## 5. Discussion

This paper has demonstrated the benefits of Economic Fitness to the policymaking process at the country, industry and sector levels. Economic Fitness is a unique contribution to the development economics literature as it provides quantitative perspectives on who is good at what and how they got there. The approach allows us to understand the past development path a country has taken and to make predictions of possible and likely paths for the future. As a rigorous and quick quantitative analysis, Economic Fitness can serve as an important tool at the beginning of the policymaking process. It is used as a filter for growth and development strategies.

While Economic Fitness carries predictive power and can initiate a country or state down a new path of development, it is important to reiterate that the Economic Fitness toolkit is not a panacea for development ills. It does not make explicit some of the critical factors that determine policy desirability or appropriateness in a given country/sector context.

**Figure 8:**
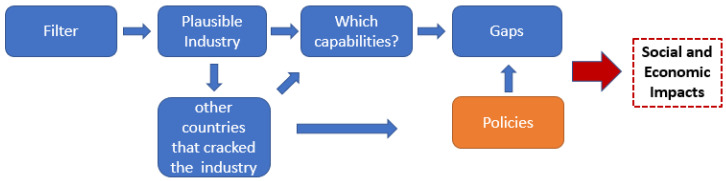
Application to the policy process. The diagram exemplifies a process for incorporating the COS framework into the policymakers’ decision process. The COS provides a filter of plausible industries. The policymaker can use Economic Fitness and other sources of information to identify which capabilities are needed to support the plausible opportunity industries and identify aspirational comparator countries. From the required capabilities and lessons from comparators, the policymaker can identify gaps within their country, state, or region, which can be targeted by appropriate policies. This can help achieve the policymakers’ goal of improved social and economic outcomes.

To achieve desired economic and social impacts, we suggest that policymakers use Economic Fitness as one of the first filters to better understand and utilize their current capabilities. Policymakers have an opportunity to use the quantitative analytics of the toolkit to think about where they want to go and assess the feasibility of getting there. The pathways of policies are specific and require traditional economic analysis. Figure 8 below can help guide policymakers through the process of understanding what it would take to achieve more economic diversification, development of green shoots or industrial capability upgrading:

The paper used the transport equipment sector as an example to illustrate the COS framework. We suggest that local policymakers and other stakeholders examine the viability and desirability of developing this industry along additional dimensions. The COS filter may help policymakers identify export subsidies that have been ineffective, because they are targeted at industries that are not likely to promote economic growth. Or we may learn about a skills gap that investment in education programs could resolve. If the cost of removing barriers to an industry is determined to be too high to be viable, we argue that this is a useful outcome of the exercise as it can eliminate one possibility and thus redirect the focus of policymakers to other options that can be more easily attained. Either way, the COS is not prescriptive of specific policies or even the level of government involvement. Accepting the view of the economy as a complex system, governments may decide to help reduce uncertainty, stabilize dynamics and provide institutional structure [28,29,30], or leave the main stabilizing force with the market [31,32,33].

## Figures and Tables

**Table 1:** Economic Fitness: What does it tell Policymakers?

Economic Fitness Toolkit Includes	But Does Not Directly Tell Us
Industry complexity (for the product, sector, country) Industry feasibility (for the product, sector, country) Export growth of an industry globally	Market demand (from domestic or export markets) Market distortions (subsidies or industry incentives available in industry of interest of elsewhere) Availability of inputs (land, labor, capital, infrastructure, export licensing and property rights regimes) Policy environment and political will Macroeconomic environment Environmental or social sustainability

**Table 2:** Name, 2015 export volume, categorical score of industry complexity (*high* classifies most complex third of industries, *mid* the second third and *low* the least complex third), progression probability (labeled feasibility), differential of 3-year compound annual growth rate relative to global rate and categorical Revealed Comparative Advantage (RCA) score (established industries have RCA > 1, green shoots have RCA < 1) for select opportunity industries in Brazil’s transport equipment sector. Source: Author calculations; Data: [25]. Brazil: Sample COS Opportunity Filter.

Description	Export Volume 2015	Industry Complexity	Feasibility	3Y Growth Rate	Type
Track-laying tractors (crawlers)	$169,653,645	mid	Already competitively exported (RCA > 1)	Growing faster than the global average	established
Bodies for tractors, buses, trucks etc	$113,090,197	high			established
Railway cars nes, open, with sides > 60 cm high	$8,493,640	high			established
Aircraft under-carriages and parts thereof	$72,305,471	high	Very feasible		Green shoot
Buses except diesel powered	$13,443,028	low	feasible		Green shoot
Railway passenger and special purpose coaches	$11,912,965	high	Slightly above average		Green shoot

**Table 3:** Name, 2015 export volume, categorical score of industry complexity (*high* classifies most complex third of industries, *mid* the second third and *low* the least complex third), progression probability (labeled feasibility), differential of 3-year compound annual growth rate relative to global rate and categorical RCA score (established industries have RCA > 1, green shoots have RCA < 1) for select opportunity industries in Mexico’s transport equipment sector. Source: Author calculations; Data: [7,25]. Mexico: Sample COS Opportunity Filter.

Description	Export Volume 2015	Industry Complexity	Feasibility	3Y Growth Rate	Type
Parts & accessories of bodies (incl. cabs) of motor vehicles	$5,313,279,076	high	Already competitively exported (RCA > 1)	Growing faster than the global average	established
Gear boxes & parts thereof	$3,861,879,366	high			established
Suspension systems & parts thereof (incl. shock-absorbers)	$1,157,224,164	high			established
Clutches & parts thereof	$315,541,609	high			established
Trucks	$11,551,733	low	Slightly above average		Green shoot
Buses	$6,075,717	low	Slightly above average		Green shoot

## Appendix A. Glossary

- **Country Opportunity Spotlight (COS).** Framework to implement the Economic Fitness toolkit and identify opportunities to raise Economic Fitness (via capability upgrade and diversification).
- **Competitive exports.** Refers in this paper to a country’s exports with RCA > 1 (see ‘Revealed Comparative Advantage’).
- **Complexity.** Exclusiveness of a good or service weighted by the inverse Fitness of producers. Conceptually, it captures the level of capabilities required to compete in an industry. Numeric score that is scaled from 0 (least complex) to 1 (most complex).
- **Capability**. The productive knowledge available in an economy that enables production of goods and services. For example, natural resources, human capital, technology, infrastructure, governance.
- **Economic Fitness.** Complexity-weighted diversification of country exports. Conceptually, it captures the level of capabilities available in an economy.
- **Economic Fitness toolkit.** Refers to the methodological framework of Economic Fitness, that is, the collection of models based on Economic Fitness. It includes Economic Fitness, Sector Fitness, Complexity, the Product Progression Network, the Progression Probability at different levels of aggregation and in different applications.
- **Established Industry.** A good or service in a country that is exported at RCA > 1.
- **Fitness**. Shorthand for ‘Economic Fitness.’
- **Fitness-GDPpc Plane.** Two-dimensional space defined by Economic Fitness (log) on the *x*-axis and per capita GDP (log) on the *y*-axis. The plane is used to model the dynamics between capabilities and income, predict growth in Economic Fitness and GDP per capita, identify comparator countries and so forth.
- **Green Shoot Industry.** A good or service in a country that is exported at RCA < 1.
- **Industry Complexity.** See ‘Complexity’
- **Middle Income Trap.** Stagnated economic growth after reaching middle income status.
- **Poverty Trap.** Stagnated economic growth among low income countries.
- **Progression Probability.** A country’s likelihood of achieving competitiveness (RCA > 1) in an industry in the next five years. A numeric score, usually normalized such that zero represents average likelihood across all countries, a positive score represents above-average likelihood and a negative score represents below-average likelihood.
- **Product Progression Network.** Network representation of goods and services, where nodes represent industries and links represent common diversification pathways. Links are based on the conditional probability of production.
- **Product Progression Taxonomy.** see ‘Product Progression Network.’
- **Regional Fitness.** Economic Fitness computed at the sub-national level, where states are treated as countries.
- **Revealed Comparative Advantage (RCA).** The share of a country’s exports in a given industry as a proportion of the country’s total exports and the total global trade in that industry as a proportion of all global trade across industries.
- **Sector Fitness.** Sectoral equivalent of Economic Fitness. Complexity-weighted diversification within a defined sector.

## Appendix B. Descriptive Statistics

**Table A1:** Economic Comparisons: Brazil and Mexico.

Indicator	Brazil	Mexico
GDP (current $bn)	1796	1046
GDP per capita (current USD)	8650	8201
population	207.7	127.5
GINI	51	48
land area	1,057,799	1,943,950
unemployment	11%	4%
consumer price inflation (end period, %)	3.90%	4.20%
government primary net lending/borrowing (% GDP)	−2.3	−2.3
current account balance (% GDP)	−1.6	−1.7

## Appendix C. Data

Gross Domestic Product (GDP) per capita. We use the World Bank definition of GDP per capita with the representation in constant international dollars and adjusted for purchasing power parity.

World Bank (2017). GDP per capita, PPP (current international $). World Development Indicators.

Product Exports: We use bilateral export data from CEPII Baci at HS6 level of disaggregation in the HS07 and HS92classification.

CEPII (2017). BACI: International Trade Database at the Product-Level.

## Appendix D. Technical Definitions

### ***Revealed Comparative Advantage (RCA)***

Revealed comparative advantage of country *c* in product *p* is defined as

RCAcp=xcp∑p′xcp′∑c′xc′p∑c′p′xc′p′

where $x_{cp}$ is the export volume of country *c* in product *p*.

### ***Economic Fitness and Industry Complexity***

The Economic Fitness *F_c_* of country *c* and the complexity *Q_p_* of product *p* are defined with the iterative formulae at each iteration *n* as:

$$F_{c}^{n}=\overset{}{\underset{p}{\sum }}M_{cp}Q_{p}^{n-1}\;$$

$$Q_{p}^{n}=\;\frac{1}{\sum_{c}^{}M_{cp}\frac{1}{F_{c}^{n-1}}}\;$$

with the initial conditions

$$\;Q_{p}^{0}=1\;$$

$$\;F_{c}^{0}=1\;$$

where *M_cp_* is the binary country-product matrix, defined as $M_{cp}=\{\begin{array}{c} 1\;if\;RCA_{cp}>1\\ 0\;if\;RCA_{cp}<1 \end{array}$ and where *RCA_cp_* is the revealed comparative advantage of country *c* in product *p*. *F* and *Q* are normalized after each iteration of the algorithm.

### ***Sector Fitness***

The Sector Fitness *F_cs_* of country *c* in sector *s* is defined over products *p* in sector *s* as above using the iterative formulae at each iteration *n*:

$$F_{sc}^{n}=\;\overset{}{\underset{p}{\sum }}S_{cp}\;Q_{p}^{n-1}\;$$

$$Q_{p}^{n}=\frac{1}{\sum_{c}^{}S_{cp}\frac{1}{F_{c}^{n-1}}}$$

with the initial conditions

$$\;Q_{p}^{0}=1\;$$

$$\;F_{sc}^{0}=1\;$$

where *S_cp_* is the binary country-product matrix over all countries *c* and products *p* in sector *s*, defined as $S_{cp}=\{\begin{array}{c} 1\;if\;RCA_{cp}>1\\ 0\;if\;RCA_{cp}<1 \end{array}$ and where *RCA_cp_* is the revealed comparative advantage of country *c* in product *p*. *F* and *Q* are normalized after each iteration of the algorithm.
